# Xenon and Argon as Neuroprotective Treatments for Perinatal Hypoxic-Ischemic Brain Injury: A Preclinical Systematic Review and Meta-Analysis

**DOI:** 10.1213/ANE.0000000000007223

**Published:** 2024-10-25

**Authors:** Mariana Barros, Min Liang, Noemi Iannucci, Robert Dickinson

**Affiliations:** From the * Anaesthetics, Pain Medicine and Intensive Care Division, Department of Surgery and Cancer, Imperial College London, London, United Kingdom; † Anaesthesiology Research Institute, Department of Anaesthesiology, First Affiliated Hospital of Fujian Medical University, Binhai Campus, Fuzhou, China; ‡ Centre for Blast Injury Studies, Imperial College London, London, United Kingdom.

## Abstract

Xenon and argon are currently being evaluated as potential neuroprotective treatments for acquired brain injuries. Xenon has been evaluated clinically as a treatment for brain ischemia with equivocal results in small trials, but argon has not yet undergone clinical evaluation. Several preclinical studies have investigated xenon or argon as treatments in animal models of perinatal hypoxic-ischemic encephalopathy (HIE). A systematic review of MEDLINE and Embase databases was performed. After screening of titles, abstracts, and full text, data were extracted from included studies. A pairwise meta-analysis of neuroprotective efficacy was performed using a random effects model. Heterogeneity was investigated using subgroup analysis, funnel plot asymmetry, and Egger’s regression. The protocol was prospectively registered on PROSPERO (CRD42022301986). A total of 21 studies met the inclusion criteria. The data extracted included measurements from 1591 animals, involving models of HIE in mice, rats, and pigs. The meta-analysis found that both xenon and argon had significant (*P* < .0001) neuroprotective efficacies. The summary estimate for xenon was 39.7% (95% confidence interval [CI], 28.3%–51.1%) and for argon it was 70.3% (95% CI, 59.0%–81.7%). The summary effect for argon was significantly (*P* < .001) greater than that of xenon. Our results provide evidence supporting further investigation of xenon and argon as neuroprotective treatments for HIE.

Hypoxic-ischemic encephalopathy (HIE) is one of the major causes of perinatal mortality and morbidity worldwide.^1^ The global incidence of HIE varies, ranging between 1 and 8 per 1000 live births in developed countries, to up to 26 per 1000 live births in developing countries. HIE results in an annual global death toll of around 1 million.^2,3^ Of those that survive HIE a high proportion (20%–40%) have severe neurological impairments that can include cerebral palsy, epilepsy, impaired motor function, loss of muscle tone, and learning deficits.^4,5^ In addition, there is evidence that survivors of HIE without major neurological impairment have impaired neurodevelopment and cognitive deficits that result in lower educational attainment.^6,7^

The standard care for moderate to severe HIE in full- or near-term neonates is therapeutic hypothermia.^8^ Although many large-scale clinical trials demonstrate that hypothermia significantly improves survival and outcomes, the degree of neuroprotection in perinatal HIE is modest while mortality and morbidity remain high, and as many as half of the cooled neonates die or have poor developmental outcome.^9–11^

In the last 2 decades, after the discovery that xenon is an *N*-methyl-D-aspartate (NMDA) receptor antagonist,^12,13^ and the identification of other pharmacological targets for xenon that may be involved in secondary injury development,^14–18^ there has been interest in the use of this noble gas as a neuroprotective treatment to prevent or limit the development of acquired brain injuries.^19–23^ Xenon’s beneficial profile may be related to pleiotropic action at multiple targets.^18,19^ Nevertheless, there is evidence that inhibition of NMDA receptors plays a significant role in xenon’s neuroprotective efficacy.^24,25,26^ In recent years, several preclinical studies have reported that the noble gas argon is neuroprotective in in vivo models of acquired brain injuries in adult animals.^27–31^ Although not as extensively investigated as xenon, a few studies suggest that argon is effective in animal models of HIE.^32–34^

**Table.** Characteristics of Included Studies

First author, year	Species, strain, sex, age/weight	Trauma model	Sample Size	Gas in treatment group	Gas in control group	Adjuvant therapy	Treatment duration	Treatment start Time	Results with treatment	Time of outcome measured after insult induction	Treatment effect (%)	Standard error
Broad et al 2016^32^	Pigs, male, aged<40 h/ 1.8–2.1 kg	Remote occlusion of both common-carotid arteries (vascular occluders) + FiO_2_ 6%–9%	18	Argon 50%	Air	HT 33.5 °C	24 h	2 h after HI	Histopathological outcome improved	2 d H	68.320	10.494
Chakkarapani et al 2010^59^	Pigs, crossbred Landrace/large white, both sexes, newborn	FiO_2_ 5%–7% on endotracheal tube for 45 min	98	Xenon 50%	70% N_2_/ 30% O_2_	HT 33.5 °C	18 or 24 h	0.5 h after HI	Histopathological and functional neurological outcome improved	3 d H/B	43.206	7.738
Dingley et al 2006^60^	Rats, Wistar, both sexses, 7 d old	Unilateral carotid ligation + FiO_2_ 8% for 90 min	45	Xenon 50%	70% N_2_/ 30% O_2_	None	3 h	Immediately after HI	Histopathological outcome improved	7 d H	82.587	18.086
Faulkner et al 2011^61^	Pigs, large-white, male, aged <24 h/1. 72 ± 0.24 kg	Remote occlusion of both common-carotid arteries (vascular occluders) + FiO_2_ 12%	32	Xenon 50%	Air	HT 33.5 °C	24 h	2 h after HI	Histopathological outcome improved	2 d H	38.289	9.331
Hobbs et al 2008^62^	Rats, both sexes, 7 d old	Common-carotid artery ligation + FiO_2_ 9% for 90 min	109	Xenon 50%	Air	HT 32 °C	3 h	Immediately after HI	Histopathological and early & long-term functional neurological improved	7 d/8–10 w B; 11 w H	25.387	11.078
Liu et al 2015^63^	Rats, both sexes, Wistar, 7 d old	Common-carotid artery ligation + FiO_2_ 8% for 90 min	37	Xenon 50%	Air	HT 32 °C	5 h	5 h after HI	Functional neurological improved, but brain area loss did not reduce	7 d/7–9 w B; 70 d H	32.777	6.234
Luo et al 2008^64^	Rats, 7 d old	Common-carotid artery ligation + FiO_2_ 8% for 210 min	18	Xenon 20% or 75%	75% N_2_/ 25% O_2_	Sevo-flurane 0.75%	2 h	4 h after HI	Histopathological and long-term neurological outcome improved; greater benefit in combination than xenon alone	7 d H; 30 d B	36.803	16.216
Ma et al 2006^65^	Rats, Sprague–Dawley, 7 d old	Common-carotid artery ligation + FiO_2_ 8% for 210 min	104	Xenon 70%	70% N_2_/ 30% O_2_	None	2 h	2, 8, or 24 h before HI	Histopathological and long-term functional neurological outcome improved	4 d H; 30 d B	74.871	5.025
Ma et al 2005^46^	Rats, Sprague–Dawley, 7 d old	Common-carotid artery ligation + FiO_2_ 8% for 90 min	202	Xenon 20%, 40%, 60% or 70%	Air	None	1.5 h	During hypoxia, or 2, 4, 6 or 24 h after HI	Histopathological and long-term functional neurological outcome improved	16 h/1 d/2 d/7 d/ 30 d H; 30 d B	43.488	4.281
Martin et al 2007^66^	Rats, Sprague–Dawley, 7 d old/10–14 g	Common-carotid artery ligation + FiO_2_ 8% for 90 min	60	Xenon 20%	Air	HT 35 °C	1.5 h	6 or 8 h after HI	Histopathological outcome improved by combination of xenon and hypothermia but not xenon alone	4 d H	12.312	3.136
Rajakumaraswamy et al 2006^67^	Rats, Sprague–Dawley, 7 d old	Common-carotid artery ligation + FiO_2_ 8% for 90 min	30	Xenon 20%	Air	Dexmedetomidine 6.25 µg/kg	1.5 h	1 h 15 min after ischemia (during hypoxia)	Histopathological and long-term neurological outcome improved in combination, but not xenon alone	4 d H; 30 d B	41.533	13.665
Sabir et al 2014^68^	Rats, Wistar, both sexes, 7 d old	Common-carotid artery ligation + FiO_2_ 8% for 90 min	185	Xenon 20% or 50%	Air	HT 32 °C or 35 °C	5 h	Immediately or 4 h after HI	Histopathological outcomes did not improve	7 d H	-1.801	3.283
Sabir et al 2016^69^	Rats, Wistar, both sexes, 7 d old	Common-carotid artery ligation + FiO_2_ 8% for 150 min	49	Xenon 50%	Air	HT 32 °C	5 h	Immediately after HI	Histopathological outcomes no improvement; weight loss no change	7 d H/W	-3.066	4.153
Sun et al 2023^70^	Rats, Sprague–Dawley, both sexes, 7 d old	Common-carotid artery ligation + FiO_2_ 8% for 120 min	45	Xenon 50%	79% N_2_/ 21% O_2_	HT 32 °C	3 h	Immediately after HI	Histopathological and functional neurological outcome improved	3/28 d H; 28 d B	36.834	9.970
Thoresen et al 2009^71^	Rats, both sexes, 7 d old	Common-carotid artery ligation + FiO_2_ 8% for 90 min	79	Xenon 50%	Air	HT 32 °C	1 h or 3 h	Immediately or 2 h after HI	Histopathological and functional neurological outcome improved	70 d H; 49–67 d B	33.162	8.142
Yang et al 2012^72^	Rats, pregnant Sprague–Dawley, 300–350 g/ Sprague–Dawley rat fetus	Uteruses were removed and placed in a water bath at 37°C for 10 min, then pups were delivered	18	Xenon 35%	70% N_2_/ 30% O_2_	None	4 h	4 h before HI	Histopathological and functional neurological outcome improved	3/7 d H; 50 d B	76.805	15.381
Zhang et al 2020^73^	Mice, C57BL/6J, 7 d old	FiO_2_ 5% for 15 min	58	Xenon 70%	79% N_2_/ 21% O_2_	None	1 h	Immediately after HI	Histopathological and functional neurological outcome improved	28 d H; 3 d/28 d/ 35 d/60 d B	67.473	3.167
Zhang et al 2019^74^	Mice, C57BL/6J, 7 d old	FiO_2_ 5% for 15 min	209	Xenon 35%, 50% or 70%	70% N_2_/ 30% O_2_	None	1 h	Immediately, 15 min or 30 min after HI	Histopathological and functional neurological outcome improved	1/3/28/60 d H; 3 d/28 d/36–40 d B	46.377	2.514
Zhao et al 2016^33^^a^	Rats, Sprague–Dawley, 7 d old	Common-carotid artery ligation + FiO_2_ 8% for 90 min	54	Argon 70%	70% N_2_/ 30% O_2_	None	2 h	Immediately after HI	Histopathological outcome improved; weight loss reduced	1/28 d H; 28 d W	71.047	14.052
Zhao et al 2016^34^^b^	Rats, Sprague–Dawley, 7 d old	Common-carotid artery ligation + FiO_2_ 8% for 90 min	70	Argon 70%	70% N_2_/ 30% O_2_	None	2 h	Immediately after HI	Histopathological outcome improved and weight loss reduced	1/28 d H; 28 d W	68.629	8.893
Zhuang et al 2012^75^	Rats, Sprague–Dawley, 7 d old	Common-carotid artery ligation + FiO_2_ 8% for 90 or 120 min	53	Argon 70%, or xenon 70%	70% N_2_/ 30% O_2_	None	1.5 h	2 h after HI	Histopathological and functional neurological outcomes improved; weight loss reduced	7 d/14 d H; 31/33–37 d B; 14/31 d W	81.799 (Ar) or 54.441 (Xe)	17.718 13.359

Ex: 1 d H; 28 d B means that the histological outcome was assessed 1 d after the insult, whereas the behavioral outcome was evaluated 28 d after the insult.

Abbreviations: B, behavioral outcome; d, days; h, hours; H, histopathological outcome; HI, hypoxia and ischemia; HT, hypothermia; min, minutes; w, weeks; weight, weight loss.

a. Oncotarget v7, p25640–51.

b Anesthesiology v125, p180.

**Figure 1.**
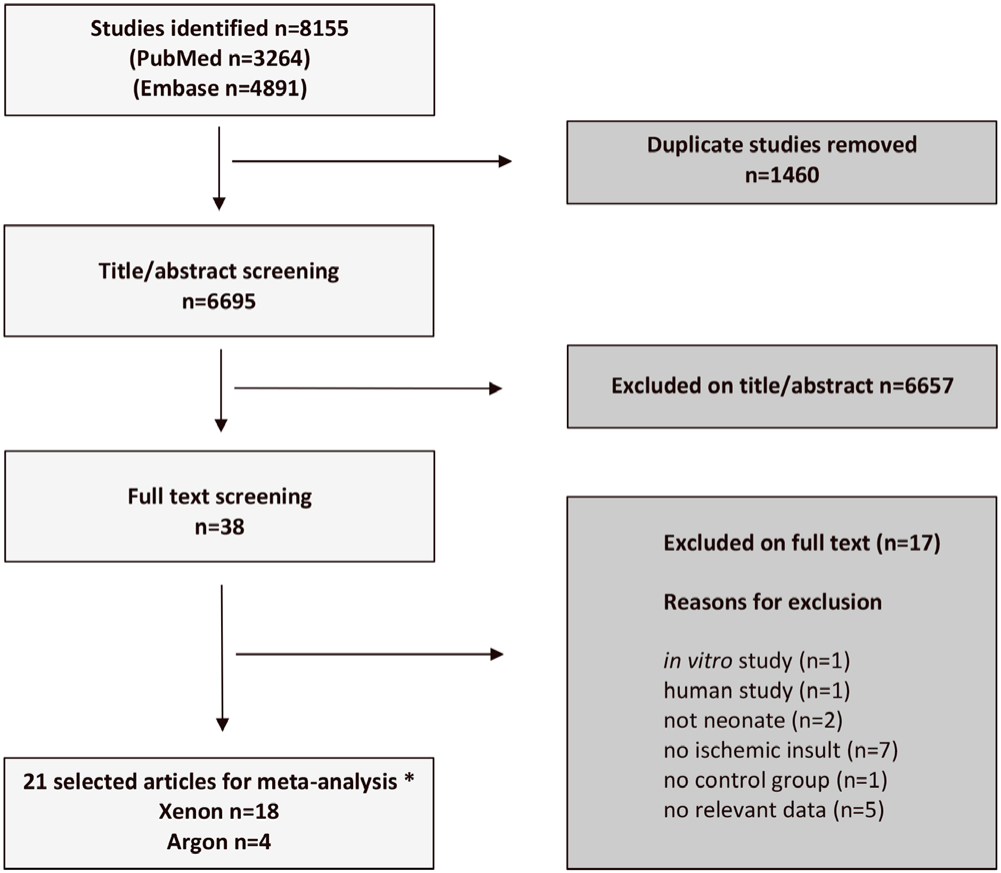
PRISMA diagram of results of systematic literature search. Twenty-one individual articles were included in the meta-analysis. One article investigated both xenon and argon. No ischemic insult: brain injury did not involve hypoxia/ischemia, No relevant data: study did not report on relevant outcome measures, for example, only physiological parameters reported. PRISMA indicates Preferred Reporting Items for Systematic Reviews and Meta-Analyses.

Until now there have not been any preclinical systematic reviews and meta-analyses that have specifically compared xenon and argon as treatments for HIE. We recently completed a systematic review and meta-analysis of xenon and argon as neuroprotective treatments for ischemic and traumatic brain injuries in adult animals,^35^ but this did not include perinatal HIE. The perinatal and developing brain is structurally and functionally different from the mature brain and its response to hypoxic-ischemic insults is different from that of the adult brain.^36,37^ Of particular relevance to the current review, the neonatal brain is more sensitive to excitotoxic insult than the adult brain, due to differential expression of NMDA receptor subunits and receptor density.^38^ In part, due to less myelination in the immature brain,^39,40^ apoptotic cell death and white matter injury are greater after hypoxia-ischemia in neonates compared to adults.^41–43^ Moreover, inflammatory and immune responses are different in neonates and adults.^44,45^ Although the mechanisms of action of xenon and argon are different, they are believed to involve several of these processes.^19,21^ In particular, NMDA receptor antagonism is involved in xenon neuroprotection against hypoxic-ischemic injury,^26^ and xenon reduces apoptosis after HI injury in neonatal rats.^46^ Xenon also reduces white matter injury in a model of traumatic brain injury through mechanisms involving modulation of neuroinflammatory glial cells.^47,48^ Argon has been shown to reduce oxidative stress *via* the transcription factor nuclear factor erythroid 2-related factor 2 (Nrf2) and heme-oxygenase-1, to reduce apoptosis, and to attenuate microglial inflammatory responses *via* mechanisms involving toll-like receptors, and nuclear factor kappa-light-chain-enhancer of activated B cells (NF-κΒ) (for a review see^21^). For these reasons, neonatal/perinatal animals represent a distinct population, and the responses to injury and to xenon or argon treatment may be different in the immature brain compared to the adult brain. The current study is a systematic literature review and meta-analysis specifically comparing xenon and argon in preclinical models of perinatal HIE. We evaluate the latest evidence about their efficacy as neuroprotective treatments for perinatal HIE to inform future preclinical and clinical research.

## METHODS

This systematic review and meta-analysis followed the guidelines of the Systematic Review Centre for Laboratory Animal Experimentation (SYRCLE) and the Collaborative Approach to Meta-Analysis and Review of Animal Data from Experimental Studies (CAMARADES).^49–51^ Our protocol was preregistered on the International Prospective Register of Systematic Reviews (CRD42022301986) https://bit.ly/3RpFxo1. In preparing the manuscript we have followed the Preferred Reporting Items for Systematic Reviews and Meta-Analyses (PRISMA) guidelines.^52^

### Research Question

The research question can be formulated in the PICO format^53^ as Population (neonatal or perinatal animals), Intervention (xenon or argon), Comparator (untreated control or with adjuvant if used), Outcome (functional neurological outcome and/or histological evidence of neuronal injury).

### Search Terms

The search terms are shown in Supplemental Digital Content 1, Supplementary Table 1, http://links.lww.com/AA/F127. Searches were implemented using the Ovid interface to access the MEDLINE (PubMed; from 1956 to 12 January 2024) and Embase (from 1947 to 12 January 2024) databases. In addition, we screened the reference lists of the included studies and articles that cited the included studies.

### Implementation of Literature Search and Screen

Three reviewers (M.B., M.L., and N.I.) independently performed the literature search and screening. After implementation of the search and manual deduplication, a title- and abstract-based screening was performed, followed by a full-text review of potentially relevant studies. Any discrepancies between reviewers in study selection were adjudicated by another researcher (R.D.).

### Inclusion and Exclusion Criteria

Detailed inclusion and exclusion criteria are described in Supplemental Digital Content 1, Supplementary Methods, http://links.lww.com/AA/F127.

### Quality Assessment

The quality of the studies was evaluated independently by 3 reviewers (M.B., M.L., and N.I.) using a modified version of the CAMARADES risk of bias checklist,^35,54,55^ see Supplementary Table 2, http://links.lww.com/AA/F127. Discrepancies between reviewers were adjudicated by another researcher (R.D.).

### Data Extraction and Transformation

Data were extracted independently by 2 reviewers (M.B. and M.L.). Another researcher (R.D.) identified any discrepancies. If the discrepancy was not resolved by independent checking by the reviewers, the third researcher decided. The information extracted and transformations are described in Supplemental Digital Content 1, Supplementary Methods, http://links.lww.com/AA/F127.

### Meta-Analysis

Pairwise and stratified random effects model meta-analyses of Normalized Mean Differences (NMDs) were performed using the CAMARADES web application (https://camarades.shinyapps.io/meta-analysis-app/) or Stata (Version 16, StataCorp), using inverse variance (1/SE^2^) weighting of individual effect sizes,^35,51,55^ and the restricted maximum likelihood (REML) estimator for tau^2^. Homogeneity among studies was quantified using the heterogeneity index (*I*^*2*^) and tested using the Q statistic. The overall meta-analysis for each gas was performed in 2 stages.^35^ In stage 1 a meta-analysis was performed for each study, giving a single overall effect size and standard error (SE) for each study. Individual effect sizes and SE for each study were then included in overall random effect meta-analyses for xenon and argon.^50,56^ Heterogeneity between studies was examined using funnel plots with the trim-and-fill method^57^ and Egger’s regression.^58^

## RESULTS

### Systematic Literature Review

The search identified 21 studies for the meta-analysis, 18 for xenon, and 4 for argon (Figure 1); 1 study investigated both gases. Details of the included studies are summarized in the Table. The animal species used were rat, mouse, and pig. Overall, data from 1591 animals was included, of which 1431 (1243 rats, 58 mice, and 130 pigs) were from xenon studies and 195 (177 rats, 0 mice, and 18 pigs) were from argon studies. The median study sizes (control, noble gas, and sham) were 56 for xenon and 54 for argon. Of note, 5 studies involved >100 animals, all of them for xenon.^46,62,65,68,74^

### Assessment of Study Quality

Using the modified CAMARADES risk-of-bias checklist, 9 of the xenon studies (50%) and 2 of the argon studies (50%) were considered high-quality low risk of bias (scores 7–9), while the other 9 of the xenon studies (50%) and 2 of the argon studies (50%) were considered as moderate quality and moderate risk of bias (scores 4–6). We did not identify any low-quality studies, with high risk of bias (scores 1–3). The overall quality scores and individual components of scores are shown in Supplementary Table 2, http://links.lww.com/AA/F127.

### Meta-Analysis. Xenon Is Neuroprotective

**Figure 2.**
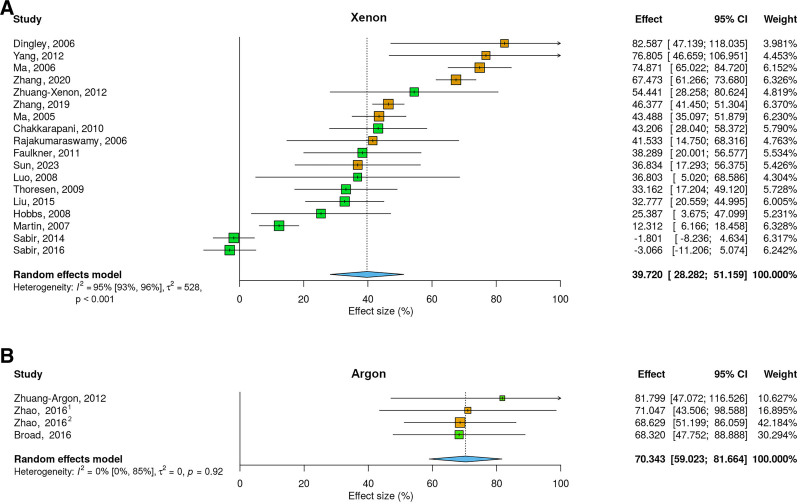
Forest plots showing estimates of effect sizes for improvement in neurological outcomes (effect size, CI, and weight) for (A) xenon and (B) argon. Positive values represent neuroprotection (improvement). The area of each square is proportional to the study’s weight in the meta-analysis. The color corresponds to study quality; high quality study with low risk of bias (green) and medium quality study with medium risk of bias (orange). The 95% Cls are shown as horizontal lines. The vertical dotted line centered on the blue diamond denotes the overall mean effect, while a vertical solid line represents no (0%) effect. The 95% CI or overall mean is represented by the width of blue diamond. The first author and date of publication are listed on the left-hand column, while the right-hand column lists the effect size, CI, and weighting for each study. ^1^Zhao et al, Oncotarget, 2016, ^2^Zhao et al, Anesthesiology, 2016. CI indicates confidence interval.

Eighteen studies investigated the neuroprotective efficacy of xenon. As shown in Figure 2A, xenon reduced neurological injury (combined histological and behavioral deficits) by 39.7% (95% confidence interval [CI], 28.3%–51.2%, *Z* = 6.8, *P* < .0001) with heterogeneity estimates *I*^*2*^ = 95% (95% CI, 93%–96%), τ^2^ = 528, Q = 473. The values for *I*^*2*^, τ^2^ and Q statistic indicate high heterogeneity in effect sizes between different studies.

Sources of heterogeneity were investigated using stratified meta-analysis. Several differences in study design were identified, including species used, xenon concentration, treatment start time and duration, and study quality parameters (eg, randomization, and blinding of injury protocol and/or outcome assessment). These differences between studies are potential sources of experimental or methodological heterogeneity.

**Figure 3.**
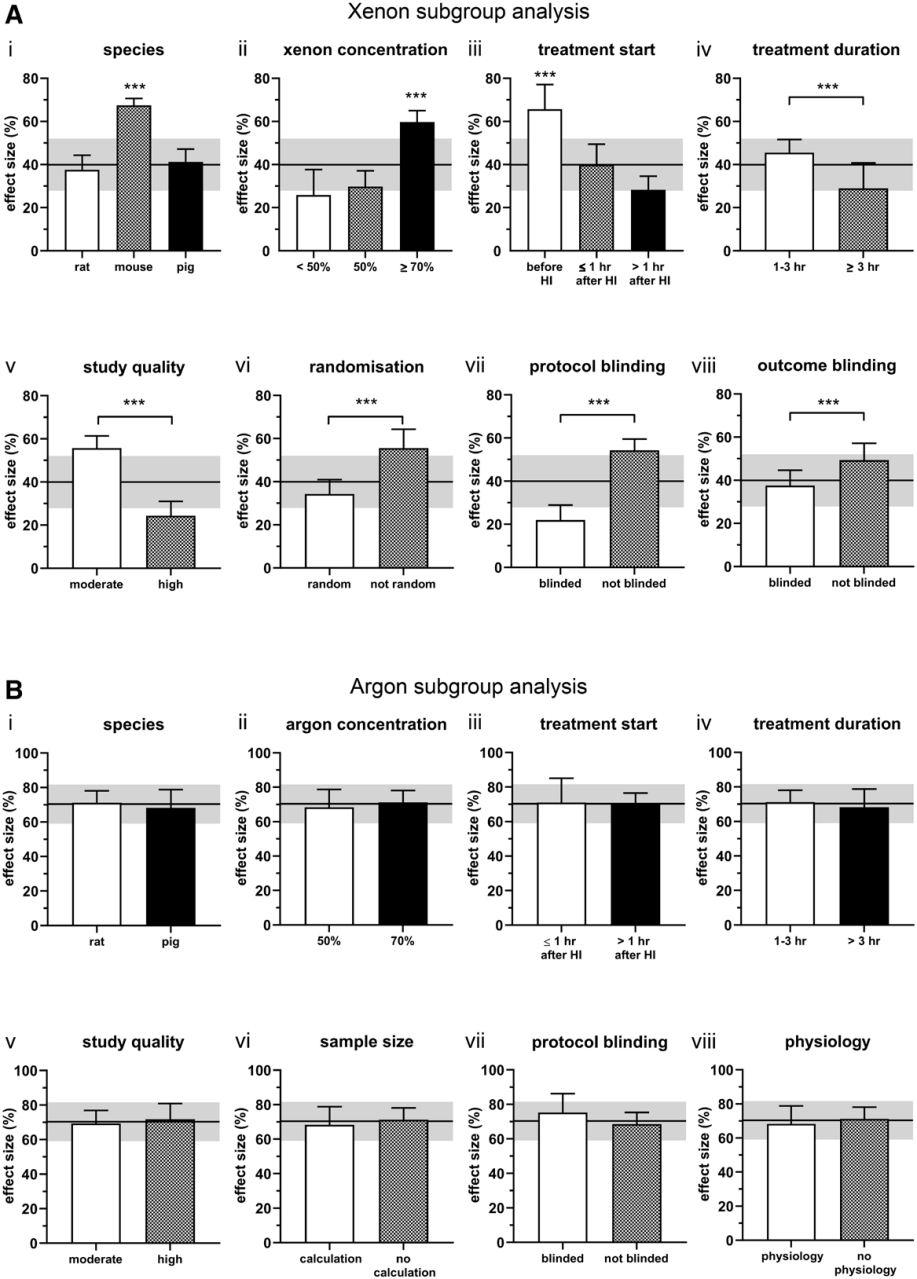
Subgroup analysis of neurological outcome effect size comparisons for (A) xenon and (B) argon. (i) Species, rat (white bar), mouse (gray bar) and pig (black bar). (ii) Gas concentration, <50% (white bar), 50% (gray bar), ≥ 70% xenon (black bar); 70% argon (black bar). (iii) Treatment start time, before hypoxic insult (HI; white bar), ≤1 h after HI (gray bar), >1 h after HI (black bar). (iv) Treatment duration 1 to 3 h (white bar), ≥3 h (gray bar). (v) Study quality, moderate (white bar), high (gray bar). (vi) Xenon, randomization (white bar), no randomization (gray bar); argon sample size calculation (white bar), no sample size calculation (gray bar). (vii) Injury protocol blinded (white bar), injury protocol not blinded (gray bar). (viii) Xenon, outcome assessment blinded (white bar), outcome assessment not blinded (gray bar); argon, physiology measured (white bar), no physiology measured (gray bar). Bars are mean values; error bars represent SEM. Differences between subgroups were tested with χ^2^ test (* *P* < .05; ** *P* < .01; *** *P* < .001). The overall meta-analysis estimate, and 95% CI are indicated by the solid gray line and the light gray shading, respectively. CI indicates confidence interval; SEM, standard error of the mean.

Subgroup analyses of the results from a stratified meta-analysis are shown in Figure 3A. Data are presented as mean (SEM). Animal species, xenon concentration, treatment start time, treatment duration, study quality, randomization, blinding of injury protocol and outcome all had a significant influence on the effect size (Figure 3Ai–viii, *P* < .001). The effect size for mouse models of 67.5% (3.2%) was larger than that for pig or rat models (Figure 3Ai, *P* < .001). The effect size was greater, 59.8% (5.2%), at the highest xenon concentration (≥70%), compared to considerably smaller values of 29.8% (7.3%) at 50% xenon and 25.9% (11.8%) at < 50% xenon (Figure 3Aii, *P* < .001). The effect size was greatest, 65.8% (11.4%), if treatment was begun before hypoxic insult, and reduced to 28.3% (6.3%) if treatment start time was delayed >1 h after hypoxic insult (Figure 3Aiii, *P* < .001). Interestingly, treatment durations of 1 to 3 hours resulted in a larger effect size of 45.6% (6.0%) than treatment durations longer than 3 h (29.0% [11.8%]; Figure 3Aiv, *P* < .001). High study quality (lower risk of bias) was associated with a smaller effect size, 24.3% (6.7%), when compared with moderate study quality with an effect size of 55.7% (5.7%; Figure 3Av, *P* < .001). As expected, lack of randomization, lack of blinding of injury protocol or outcome measures resulted in greater effect sizes (Figure 3Avi–viii, *P* < .001). A subgroup analysis by the institution where studies were performed (Supplementary Figure S1 http://links.lww.com/AA/F127) showed there were significant differences (*P* < .001) between institutions, with Bristol University having the lowest effect size (27.5% [10.0%]) and Binzhou Medical University reporting the largest effect size (56.8% [10.5%]).

**Figure 4.**
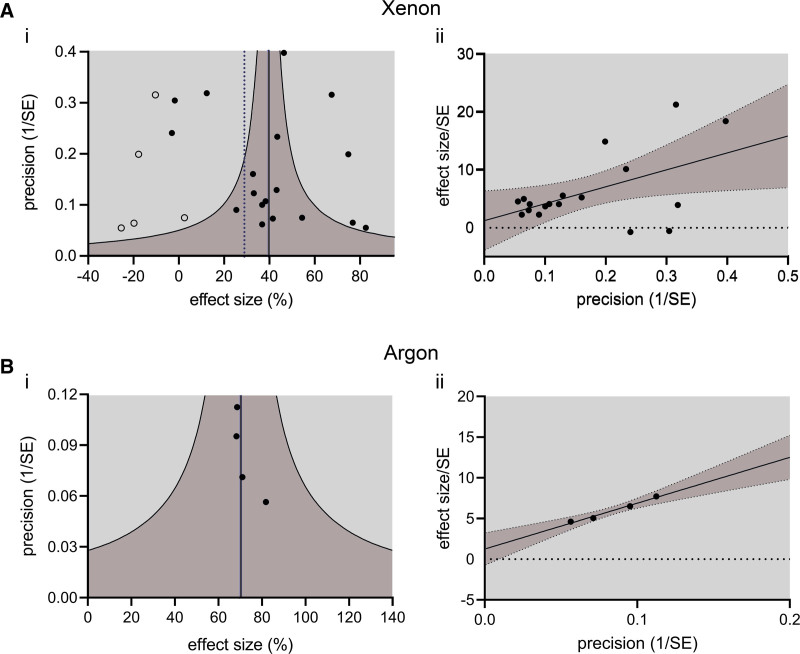
Heterogeneity analysis of the neuroprotection studies. (A) (i) Funnel plot for xenon. Trim-and-fill analysis detected asymmetry, and 5 imputed studies were suggested. (ii) Egger’s regression analysis for xenon studies. The *y*-axis intercept of 1.3 ± 2.6 was not significantly different (*P* = .63) to zero indicating no asymmetry. (B) (i) Funnel plot for argon. Trim-and-fill analysis detected no asymmetry, and no imputed studies were suggested. (ii) Egger’s regression analysis of the argon studies. The *y*-axis intercept of 1.2 ± 0.5 was not significantly different (*P* = .11) to zero indicating a no asymmetry. In the funnel plots study effect size is plotted on the *x*-axis, the reciprocal of the standard error, as a measure of study precision, is plotted on the *y*-axis. Vertical black solid line represents the meta-analysis summary effect sizes and the dashed vertical lines represent estimates including imputed studies, where present. Dark gray shaded area within curved lines represents 95% CI for the random effects model. Filled circles represent actual data points, open circles are imputed studies from trim-and-fill analysis of xenon funnel plot. In Egger’s regressions the *x*-axis is the reciprocal of the standard error, and the *y*-axis is the ratio of effect size to standard error. The line is the central estimate and dark gray shading represents the 95% CI. CI indicates confidence interval; SE, standard error.

Trim-and-fill analysis of a funnel plot identified asymmetry and suggested 5 (3) imputed studies on the left side, as shown in Figure 4Ai. A possible explanation of the funnel plot asymmetry is publication bias whereby studies with small or insignificant effects are missing. The estimated effect size including the imputed studies was 28.9% (95% CI, 16.2%–41.6%), *I*^*2*^ = 96%, *P* < .001, a 10.8% reduction compared with the originally observed value, 39.7%. Interestingly, heterogeneity was not evident in Egger’s regression analysis, where the intercept was not significantly different to zero (intercept = 1.2 (2.4), *P* = .62; Figure 4Aii).

### Stratified Meta-Analyses of Outcome Type and Outcome Timepoint Meta-Regression

The overall meta-analysis above combined histological and behavioral outcomes into a global measure of neurological injury. The rationale for this strategy is that not all studies included all types of outcomes and a global measure would utilize contributions from the maximum number of studies, although there is the possibility that combining outcomes could result in heterogeneity. To investigate the validity of our approach, we performed a stratified meta-analysis of the xenon studies (n = 18) where we separated out each of these outcomes (Supplementary Figure 2 http://links.lww.com/AA/F127. The overall effect size for histology, 43.9% (95% CI, 30.8%–56.9%; 18 studies) was larger than that for behavior, 38.5% (95% CI, 25.9%–51.0%; 13 studies), but the 95% CIs overlapped with each other and with the effect size of combined analysis of 41.6% (95% CI, 32.4%–50.8%).

In addition, in our analyses we pooled outcome data at different time points. For the xenon studies the outcome measurement times were heterogeneous and ranged from 16 hours to 11 weeks after HI injury. To investigate the effect of outcome measurement time, we performed a meta-regression of the effect size of each outcome with respect to the time of the assessment (in days) after the HI injury (Supplementary Figure 3 http://links.lww.com/AA/F127). The slope of the regression line was not significantly different (*P* = .73) to zero, indicating the time of the outcome assessment in these studies did not have a significant effect on outcome effect size.

### Meta-Analysis: Argon Is Neuroprotective

We identified 4 studies that investigated the effects of argon. As shown in Figure 2B, argon reduced neurological injury (combined histological and behavioral deficits) by 70.3% (CI, 59.0%–81.7%), *Z* = 12.2, *P* < .0001 with heterogeneity estimates of *I*^*2*^ = 0% (95% CI, 0%–85%), τ^2^ = 0 and Q = 0.49.

The values for *I*^*2*^, τ^2^ and Q statistic indicate low heterogeneity in effect sizes between the different studies. Sources of heterogeneity were explored using stratified meta-analysis. The subgroup results are shown in Figure 3B and Supplementary Figure S1 (data presented as mean [SEM]). None of the subgroups investigated (species, argon concentration, treatment start time or duration, study quality, sample size calculation, and blinding of injury protocol, physiological measurements or institution) had a significant effect on the magnitude of the effect size (Figure 3Bi–viii and Supplemental Digital Content 1, Supplementary Figure S1, http://links.lww.com/AA/F127).

Trim and fill analysis did not identify asymmetry in the funnel plot and no imputed studies were suggested, as shown in Figure 4Bi. In agreement with this, Egger’s regression (Figure 4Bii) did not suggest the presence of asymmetry, with the intercept not significantly different to zero (1.25 [0.46], *P* = .11).

### Meta-Analysis: Argon Is More Neuroprotective Than Xenon

**Figure 5.**
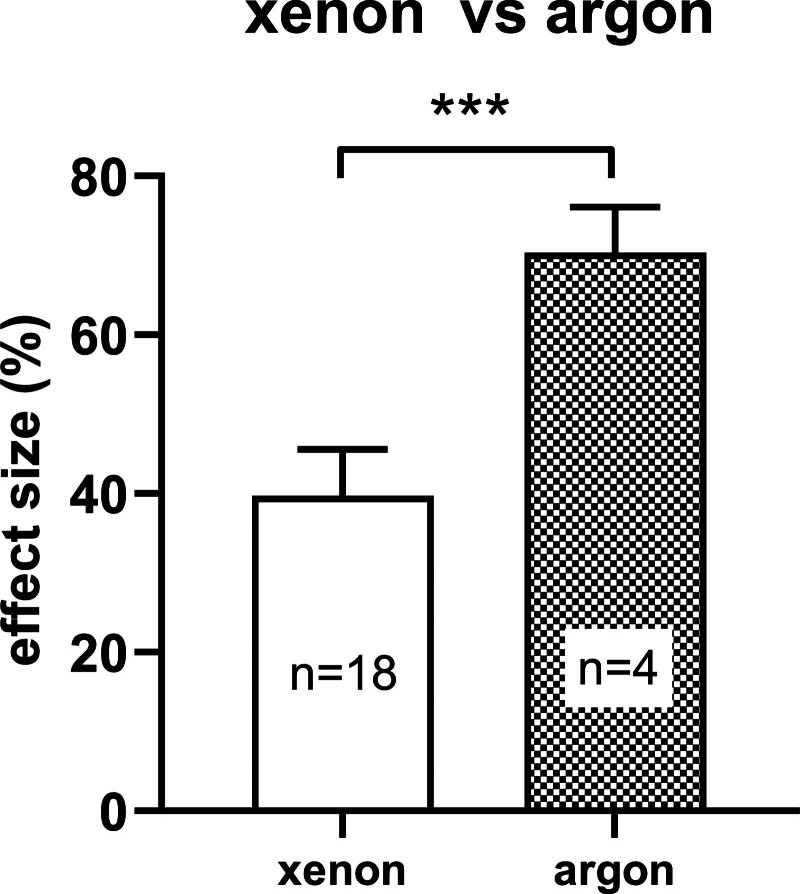
Comparison of overall neuroprotective effects of xenon (white bar) and argon (gray bar) in perinatal hypoxic-ischemic brain injury. Bars represent the mean effect size and error bars represent SEM. ***P < .001 χ^2^ test. The number of studies are as follows: n = 18 for xenon, and n = 4 for argon. SEM indicates standard error of the mean.

Finally, we compared the efficacy of xenon and argon with a global stratified meta-analysis of all 22 studies with “gas treatment” as a categorical variable (Figure 5). The overall efficacy of argon, 70.3% (95% CI, 59.0%–81.7%) was significantly (*P* < .001) greater than that of xenon, 39.7% (95% CI, 28.3%–51.2%).

## DISCUSSION

As far as we are aware, this is the first preclinical systematic review and meta-analysis to directly compare argon and xenon as neuroprotectants specifically in perinatal HIE. Several reviews of argon and xenon as general brain or organ protectants have been published.^20,21,76–78^ A few narrative preclinical reviews have specifically focused on treatments for perinatal HIE including argon and xenon.^79–83^ An earlier preclinical systematic review and meta-analysis of noble gases as treatments for ischemic injury in brain and other organs included perinatal HIE, but pooled this data with adult cardiac arrest and stroke models^84^ that, as discussed earlier, represent distinctly different populations and insults.

### Systematic Review

We identified 21 studies, published between 2005 and 2023, for inclusion in the meta-analysis. More studies reported on xenon (n = 18) than reported on argon (n = 4) and 1 study reported on both gases. Study quality, assessed with modified CAMARADES checklist, indicated 10 studies of high quality (low risk of bias) and 11 of medium quality (medium risk of bias). The proportion of high-quality studies is lower than in our previous meta-analyses in adult animals but higher than other preclinical meta-analyses.^35,85^

### Meta-Analyses

The main finding of the meta-analyses for xenon and argon is that the summary effect sizes were greater than zero with 95% CIs that do not include zero, indicating significant (*P* < .0001) improvement in histological and neurological outcomes for both gases compared to untreated groups.

### Xenon

The overall summary effect size for xenon was 39.7% (95% CI, 28.3%–51.2%), *P* < .0001. The significant overall neuroprotection that we observed for xenon in perinatal HIE is consistent with our previous finding in a meta-analysis of adult animal models of cardiac arrest (global ischemia), ischemic stroke and TBI.^35^ In that study^35^ we found an effect size for xenon of 34.1% (95% CI, 24.7%–43.3%), *P* < .0001, similar in magnitude to what we find here for perinatal HIE. Our current results are qualitatively consistent with those of De Deken et al^84^ who found significant neuroprotection with xenon in combined adult and perinatal ischemic brain injury in rats and mice.

The trim and fill analysis of the funnel plot asymmetry is one that seeks out “missing studies” resulting from publication bias. Trim and fill analysis of the xenon perinatal HIE studies suggested 5 (3) imputed studies on the left-hand side (lower effect size) of the funnel plot that, if included, would reduce the overall summary effect. In contrast to this, Egger’s regression did not find asymmetry. Taking these conflicting results together, it is unclear whether there is true asymmetry; a more detailed discussion of potential sources of heterogeneity is given below.

### Argon

The overall summary effect size for argon was 70.3% (95% CI, 59.0%–81.7%), *P* < .0001. While the number of argon studies is low, our finding of significant overall neuroprotection by argon is consistent with our previous report for argon in adult animals, in models of cardiac arrest (global ischemia), ischemic stroke and TBI.^35^ Interestingly, the magnitude of the effect size in perinatal HIE is greater than the 18.1% (95% CI, 8.1%–28.1%), *P* < .001, we previously found in adult animal models of acquired brain injury.^35^ These findings contrast with the earlier meta-analysis of De Deken et al^84^ that reported no significant protection by argon in ischemic brain injury in both adult and perinatal rats and mice. However, the study by De Deken et al^84^ was performed in 2016 and included only 1 argon perinatal HIE study, that of Zhuang et al.^75^ Since De Deken et al^84^ was published, an additional 3 studies of argon in perinatal HIE have been published and these are included in our current study.^32–34^ In addition, as we discussed previously,^35^ although the 95 % confidence intervals in De Deken et al^84^ overlapped zero (hence the lack of significance at *P* < .05), the summary standardized effect size was positive.

Trim and fill analysis of the funnel plot of all argon perinatal HIE studies did not find asymmetry or suggest imputed studies. Consistent with this, Egger’s regression did not detect asymmetry.

### Heterogeneity

The meta-analyses identified high heterogeneity in the xenon studies with an I^2^ = 95% (95% CI, 93%–96%). Of note are the high heterogeneity index and the tight confidence intervals around this estimate. Therefore, we provide strong evidence for the presence of heterogeneity in the xenon meta-analysis that is unlikely to be due to sampling errors or publication bias (“missing studies”), but more likely due to differences in the study methodologies (see discussion below). We used a random effects model because it acknowledges heterogeneity between studies that is common in preclinical meta-analyses and provides a more generalizable estimate of the effect size with wider (more conservative) confidence intervals.^51,86^ The observed high heterogeneity supports our choice of a random effects model for the meta-analysis.

In contrast with the findings for xenon, for the argon studies the heterogeneity estimate was I^2^ = 0% (95% CI, 0%–85%). At first sight a low I^2^ might be expected where the individual study effect sizes are very close together. Nevertheless, while the central estimate is zero, the confidence interval is very wide, with a maximum value of 85%, meaning that the true I^2^ could be anywhere between 0 and 85%. In this particular case it is likely that the wide confidence interval can be attributed to the low number of studies (n = 4) included in the argon meta-analysis, making it difficult to be certain whether or not there is heterogeneity in the argon meta-analysis.^87^

In preclinical research there are often fundamental differences in experimental design and outcome measures used by different laboratories. Given that we are comparing different animal studies, high heterogeneity values are expected, similar to those observed in other preclinical meta-analyses.^35,84^ For example, to increase the generalizability of our findings we included different species (mouse, rat and pig) in which there may be differing underlying pathophysiology.

Reporting bias or publication bias, commonly stated as preferential publishing of positive findings, is another possible source of the heterogeneity.^58^ The trim-and-fill analysis suggested that the estimate of the xenon summary effect size was enhanced and suggested 5 imputed (or “missing”) studies. However, the Egger’s regression did not identify asymmetry in the xenon group, suggesting that there are no missing publications. In the case of xenon, the asymmetry in the funnel plot is likely to be explained by the heterogeneity arising from differences in study methodologies. Nevertheless, publication bias cannot be entirely ruled out.^88^

### Subgroup Analysis

In addition to explaining sources of heterogeneity, subgroup analyses may yield a more precise estimate of effect size under specific conditions (eg, species, or particular drug dose). It is important to note that if the number of studies in the meta-analysis is small, subgroups may have few studies, making the subgroup analysis underpowered to detect differences.^50^

Nevertheless, some interesting findings were observed. In the xenon analysis, significant differences were observed for animal species. Mouse models had a higher mean effect size than rat or pig models, outside the 95% confidence interval of the overall summary estimate. However, piglet and rat models had very similar effect sizes that were both close to the overall effect size. A caveat to the interspecies comparisons is that within a given species, different genetic strains have been used (eg, Wistar & Sprague Dawley rats) that are an additional source of heterogeneity. Xenon concentration resulted in significant differences in effect size, with the highest concentration used, ≥70%, resulting in a greater effect size than 50% or less xenon, as expected from a classical pharmacological dose-response. Treatment start time also resulted in significant differences in effect size, with the greatest effect size, outside the 95% confidence interval of summary estimate, occurring in the situation where the treatment was begun before the hypoxic insult; the next highest effect size occurred when treatment was started 1 hour or less after injury, whereas the smallest effect size was when treatment was started at times greater than 1 hour after injury. Although maximum effect size with pretreatment is to be expected, giving the treatment before the brain injury is a scenario with limited clinical applicability. Nevertheless, the trend in effect size with respect to treatment initiation time serves to emphasize that the clinical objective should be to initiate treatment as soon as possible after the HI insult. Treatment duration also resulted in significant differences in effect size, with the larger effect size being with shorter treatment durations of between 1 and 3 hours compared to durations of 3 hours or longer, although both effect sizes were within the 95% confidence interval of the summary effect size. Although this result appears paradoxical, an explanation may be found in covariates. Of the 6 studies with treatment durations of greater than 3 hours, 5 apply hypothermia as a cotreatment, 2 are in pig models and 4 are in rats, and all of these studies used xenon concentrations of 50% or less. The reason why pigs and rats were used in the longer duration studies may be that prolonged hypothermia is technically easier to implement in these species compared to mice. Hence the shorter duration group contains more mouse models combined with xenon concentrations of 70% or greater, both of which are associated with larger effect sizes. In addition, the studies with longer treatment duration tended to be higher quality (5 of 6 high quality), than those with shorter treatment duration (4 of 12 high quality). It is to be expected that higher quality (lower risk of bias) studies would have more conservative (smaller) estimates of effect size than those of lower quality, because higher quality studies are less likely to have confounders such as lack of randomization or blinding that could lead to overestimates of efficacy. This is borne out in the observation that of all the included studies, the moderate quality studies resulted in significantly higher effect size than high quality studies, and by our finding that lack of randomization or lack of blinding of the injury protocol and/or the outcome measures all resulted in significantly higher effect sizes. These results are consistent with the validity of study quality measures included in the CAMARADES checklist.^35,54,55^ In the case of the argon meta-analysis the fact that none of the subgroups analyzed resulted in a significant difference in effect size supports a lack of heterogeneity, but a caveat is that the total number of studies is small (n = 4).

### Stratified Meta-Analysis and Meta-Regression

In the stratified meta-analysis we investigated the effect of histological and behavioral outcome measures on the effect size. A criticism of some early animal studies of ischemic stroke was that a focus on histological outcomes rather than functional outcomes is less translationally relevant because histological improvement may not map to behavioral improvements.^89^ This has been cited as a potential explanation for the failure of some treatments to demonstrate efficacy in clinical trials and led to the recommendation that preclinical studies include both histological and behavioral outcomes.^89–91^ The majority (72%) of included studies have both types of outcomes, allowing us to compare them. Our results showed that while the mean effect size for histological outcomes was larger than for behavioral outcomes, the 95% CIs overlap with each other, and with the combined estimate. This suggests that, on average, histological outcomes are a reasonable surrogate for functional outcomes, and justifies our decision to pool data from these outcomes into a composite measure of neuroprotection.

It is accepted that longer term outcomes in preclinical studies are more relevant than short-term acute outcomes when compared to the clinical scenario. However, until relatively recently, few animal studies have investigated very long-term or chronic timepoints. Our review identified studies with outcomes assessed from 16 hours up to 70 days after HI insult. Interestingly, the meta-regression found that there was no significant effect of time on observed effect size. This finding was unexpected because in adult animal injury models larger effect sizes are typically observed at very early time points compared to chronic or long-term outcomes. However, a caveat is that in the case of these perinatal HIE models, the 70-day timepoint represents juvenile/young adult animals, and we did not identify any studies that included very long term or chronic follow-up times.

### Comparison of Xenon and Argon

The main finding of this work is that significant neuroprotective efficacy was observed with both xenon and argon. We identified 18 studies on xenon and 4 studies on argon. If we compare xenon and argon in the same meta-analysis with treatment gas as a subgroup, we find that the efficacy of argon 70.3% (5.8%) is significantly (*P* < .001) greater than the efficacy of xenon, 39.7% (5.8%). However, an important caveat is that the number of argon studies in HIE models is small, and additional studies would be helpful, ideally directly comparing the 2 gases. Nevertheless, the finding of greater efficacy for argon compared to xenon in perinatal HIE contrasts with our previous meta-analysis of preclinical models of acquired brain injury^35^ in adult animals where xenon was significantly more efficacious than argon. Interestingly, although De Deken et al^84^ did not find significant argon neuroprotection for ischemic brain injury in adult and perinatal mice and rats, the mean standard effect size values were greater for argon than for xenon. Limitations are discussed in Supplemental Digital Content 1, Supplementary Information, http://links.lww.com/AA/F3.

### Clinical Relevance

The neuroprotective efficacy of xenon has been investigated in a few early-stage clinical trials of ischemic brain injury: in perinatal HIE and brain ischemia caused by out-of-hospital cardiac arrest in adults.^92–94^ The perinatal HIE trial enrolled 92 babies and found no effect on the primary outcomes (MRS assay of lactate to N-acetyl aspartate ratio and MRI fractional anisotropy as surrogates of brain injury).^92,95^ This negative result may be explained by the long delay to start xenon treatment, median 10.0 hours (IQR 8.2–11.2),^92^ that is likely to be outside of the therapeutic time window (between 3 hours, and 6 hours in preclinical studies).^63,96^ An adult trial with 110 out-of-hospital cardiac arrest patients reported a positive neuroprotective effect on its primary and secondary outcomes of preservation of white and gray matter, respectively (assessed by MRI fractional anisotropy and structural T1-weighted imaging).^93,94^ An important difference in this study was the shorter time to start the xenon treatment, median 4.1 hours (IQR 3.4–4.6). This serves to illustrate the importance of determining therapeutic time window for xenon in preclinical and clinical studies. Xenon is more expensive than argon and requires the use of a closed circuit. Nevertheless, if xenon is shown to improve long-term clinical outcomes in brain injury patients, then this could justify the cost of xenon and associated delivery hardware. Currently, no clinical trials in neonates or adults assessing argon’s neuroprotective efficacy in any type of acquired brain injury have been reported. Our current preclinical meta-analysis supports future clinical studies of xenon and argon in HIE, but with the strong caveat for argon that due to the low number of studies, further preclinical research is merited.

## CONCLUSION

Our meta-analysis found both xenon and argon are neuroprotective in preclinical models of perinatal HIE, with argon providing significantly greater neuroprotection than xenon from current evidence. Our findings should inform future preclinical and clinical study protocols. Further preclinical studies with both gases are necessary to determine the time window of efficacy in perinatal HIE, and it is recommended that additional preclinical studies demonstrating the efficacy of argon are performed before proceeding to clinical trials.

## DISCLOSURES

**Conflicts of Interest:** R. Dickinson has received funding for research on xenon neuroprotection from the funding bodies below. No other authors declared Conflicts of Interest. **Funding:** This work was supported by the Medical Research Council, London, United Kingdom (MR/N0277361/1); Association of Paediatric Anaesthetists of Great Britain & Ireland; Royal Centre for Defence Medicine, Birmingham, United Kingdom; Centre for Blast Injury Studies, Imperial College London; and Royal British Legion. **This manuscript was handled by:** Peter A. Goldstein, MD.

## Supplementary Material


