# Supplementary material for: Xenon and Argon as Neuroprotective Treatments for Perinatal Hypoxic-Ischemic Brain Injury: A Preclinical Systematic Review and Meta-Analysis
Source: Anesth Analg. 2024 Oct 25;141(2):327–42. doi: 10.1213/ANE.0000000000007223 (PMC12220577; doi:10.1213/ANE.0000000000007223)
Supplement: Supplementary file 1 [file ane-141-327-s001.docx]

**Methods**

***Inclusion criteria.*** Eligible publications were preclinical studies on the effects of xenon or argon in neonatal or perinatal HIE, in English. There were no restrictions on publication year, time of initiation or duration of treatment, or concentration of the therapeutic gas. The selected publications, (i) investigated neuroprotection in neonates treated with either xenon or argon, (ii) included as outcomes (a) neurological function and/or (b) lesion volume or neuronal injury; (iii) included a control group, such that the only variation was the treatment gas. In studies that combined noble gas treatment with another therapy, such as hypothermia, the control group was considered this treatment alone if such a group was included, and (iv) with or without a sham group.

***Exclusion Criteria.*** Studies were excluded if they (i) were in humans; (ii) were *in vitro* studies, (iii) used adult animals; (iv) did not investigate xenon or argon as neuroprotectants; (v) lacked required data for meta-analysis (e.g. group sizes) and this data could not be obtained from the authors; or (vi) lacked the required outcome measures, e.g. only physiological data reported. We did not include neuroinflammatory outcomes in the meta-analysis, because changes in inflammatory markers are difficult to interpret in terms of neuroprotection (e.g. microglia may be neuroprotective or neurotoxic depending on their activation state and an increase in total number of microglia may have opposing effects depending on activation state).

***Data extraction and transformation****.* Data were extracted independently by two reviewers (MB, ML). Another researcher (RD) identified any discrepancies. If the discrepancy was not resolved by independent checking by the reviewers, the third researcher decided. The information extracted was: (i) neurological function including motor, and cognitive tests, (ii) histological evaluation, including infarct volume, neuronal count or density, and number of degenerating or apoptotic neurons, and (iii) body weight change, as the degree of early weight loss correlates with injury severity in animal models of brain injury. Data that could not be unambiguously related to neurological injury or neuroprotection were not included (e.g. physiological data or neuroinflammation data). For each of the outcomes the following data were extracted: mean (X), standard error of the mean (SEM), standard deviation (SD) and the total number of animals per group (n). If the same control group is used for more than one treatment group then the ‘true number of control animals ($n_{c}^{'})$’ was calculated from equation (1) ^1^

$n_{c}^{'}=\frac{n_{c}}{Treatment group served by one control}$ (1)

Where numerical values of outcomes were reported these were recorded directly. In the case of graphical presentation, numerical data were extracted from digitised images of graphs using a plot digitiser tool (<https://automeris.io/WebPlotDigitizer/>). Extracted results were transformed into a format compatible with the CAMARADES web-based meta-analysis application (see user guide <https://bit.ly/3EB4mFX>). When median, range or interquartile range were reported, mean and SD were calculated using the median, range and sample size. ^1,2^ If behavioral neurological outcomes were tested more than once at different times, data from each time point was included in the meta-analysis for the paper, representing an overall measure of that outcome for each cohort. ^3^ In addition, the concentration and type of noble gas, animal species, time of initiation of gas treatment, duration of gas treatment, and the overall conclusions of the study were recorded.

The normalized mean difference (NMD) effect size estimate was used because it allows outcomes on different measurement scales to be analysed in the same meta-analysis. The NMD calculation is shown in equation (2), where x_c_ is the mean value of control group, x_sham_ is the mean value of sham group and x_rx_ is the mean value of treatment group)

$NMD=100\%\times\frac{\left( \overline{x}_{c}-\overline{x}_{sham} \right)-\left( \overline{x}_{rx}-\overline{x}_{sham} \right)}{\left( \overline{x}_{c}-\overline{x}_{sham} \right)}$ (2)

In preclinical studies where the sample size may be small, random error or experimental model design can result in the observed injury effect (difference between control injury and sham) being modest. Hence the denominator in equation (2) may be very small and this can lead to extreme positive or negative NMD values. We used the method proposed by Vesterinen *et al* ^3^ to deal with cases where the absolute value of effect size calculated using equation (2) was greater than 100%, for further details see Liang *et al.* ^1^

***Meta-analysis Heterogeneity****.* Heterogeneity between studies was examined using funnel plots with the trim-and-fill method and Egger’s regression. Potential sources of heterogeneity were further investigated using stratified meta-analyses. Predefined potential sources of heterogeneity were animal species, sex, study quality, sample size calculation, randomization, blinding to assessment of outcome, temperature control and inclusion of sham group. In addition, we investigated gas concentration, treatment start time and treatment duration. Subgroup differences in stratified meta-analyses were tested with a chi-square test.

***Limitations.***  Animal models of brain injury have been playing an important role in the evaluation of novel treatments for brain injury. However, preclinical models have limitations related to clinical translation. In animal studies an added complexity is defining what a ‘clinically meaningful’ effect size is for a measure such as reduction in infract volume or number of degenerating neurons. The ages of all the animals at the time of injury and treatment ranged from before birth (rat fetus model) to <24hr – 40-hr after birth (piglet models) to 7 days after birth (rodent models). The 7-day rodent models are the most widely used preclinical HIE model and most of the studies used 7-day old rodents. However, this represents a slightly different developmental stage to neonatal piglets (and humans). The use of different animal species is an obvious complexity. Rodent models have the advantage of lower cost, a large array of well validated behavioral tests and the ability to investigate chronic effects on a feasible timescale. In the case of models of HIE the common use of rodents results in specific technical difficulties compared to larger animals. Due to their small size, it is technically challenging to intubate and ventilate rodent pups meaning that most studies rely on spontaneous breathing during the treatment phase (and in some cases during hypoxic insult). It is technically challenging to monitor oxygen saturation, or even respiratory rate, in rodent pups. As a result, there may be unintended apnea and hypoxia in these models that would confound both severity of insult and the treatment paradigms. In this respect piglet models are better because ventilation and monitoring are easier to facilitate. An additional advantage of pig models is that, like humans, they have a gyrencephalic brain whereas the rodent brain is lissencephalic. However, piglet models are more complex, expensive and usually implemented with only short-term outcomes (hours to 1 or 2 days). In our meta-analysis there were 3 piglet studies, 2 for xenon and 1 for argon. It is recognized that from a clinical perspective, outcomes in neuroprotection experiments should ideally include long term or chronic timepoints. An advantage of rodent models is that longer term outcomes are more easily facilitated. Of the 19 studies using rats or mice, 14 had one or more outcome measure at 28 days or longer after injury.

A limitation of the comparison of the efficacy of xenon and argon is that it is dependent on meta-analyses of a different studies from different laboratories, involving different species as discussed above. To definitively determine whether one or other noble gas is more effective, ideally both gases should be compared in the same study under identical conditions. Only one study, that of Zhuang *et al* ^4^ , directly compared the two gases. Interestingly, this study reported that argon was more effective (81.7% protection), compared to xenon (54.4% protection) that mirrors the result of our meta-analyses.

An aspect that we were unable to fully address is the important question of the therapeutic time window after injury during which the treatments are effective. Only 3 studies (in the xenon group) specifically investigated treatment start time as an experimental variable. ^5,6,7^ Nevertheless, although in the xenon group efficacy decreased as the time delay to treatment initiation increased, it was not possible to determine a cutoff time when treatment was ineffective. This is a key aspect from the clinical translation perspective, that merits further investigation for both gases.

An additional source of heterogeneity is that many of the studies included an ‘adjuvant therapy’ in addition to argon or xenon. In most cases this adjuvant was therapeutic hypothermia because this is the current clinical treatment for HIE, but one study included dexemedetomidine and another sevoflurane. In cases where an adjuvant was used the appropriate control used was the adjuvant alone, if this group was present, so that we were analysing the effect size of the noble gas alone. This procedure will take account of additive effects, but in the case of synergistic interactions it may over-estimate the effect of the noble gas.

**References**

1. Liang M, Ahmad F, Dickinson R. Neuroprotection by the noble gases argon and xenon as treatments for acquired brain injury: a preclinical systematic review and meta-analysis. Br J Anaesth 2022; **129**: 200-18
2. Hozo SP, Djulbegovic B, Hozo I. Estimating the mean and variance from the median, range, and the size of a sample. BMC Med Res Methodol 2005; **5**: 13
3. Vesterinen HM, Sena ES, Egan KJ, Hirst TC, Churolov L, Currie GL, Antonic A, Howells DW, Macleod MR. Meta-analysis of data from animal studies: a practical guide. J Neurosci Methods 2014; **221**: 92-102
4. Zhuang L, Yang T, Zhao H, Fidalgo AR, Vizcaychipi MP, Sanders RD, Yu B, Takata M, Johnson MR, Ma D. The protective profile of argon, helium, and xenon in a model of neonatal asphyxia in rats. Crit Care Med 2012; 40: 1724-30
5. Ma D, Hossain M, Chow A, Arshad M, Battson RM, Sanders RD, Mehmet H, Edwards AD, Franks NP, Maze M. Xenon and hypothermia combine to provide neuroprotection from neonatal asphyxia. Ann Neurol 2005; 58: 182-93
6. Sabir H, Walloe L, Dingley J, Smit E, Liu X, Thoresen M. Combined treatment of xenon and hypothermia in newborn rats--additive or synergistic effect? PLoS One 2014; 9: e109845
7. Luo Y, Ma D, Ieong E, Sanders RD, Yu B, Hossain M, Maze M. Xenon and sevoflurane protect against brain injury in a neonatal asphyxia model. Anesthesiology 2008; 109: 782-9

**Supplementary Table 1. Search strategy**

| #1 | xenon |
| --- | --- |
| #2 | argon |
| #3 | noble gases OR inert gases |
| #4 | #1 OR #2 OR #3 |
| #5 | newborn |
| #6 | infant |
| #7 | pups |
| #8 | neonat* |
| #9 | perinat* |
| #10 | #5 OR #6 OR #7 OR #8 OR #9 |
| #11 | hypoxi* |
| #12 | ischemi* |
| #13 | ischaemi* |
| #14 | asphyxi* |
| #15 | encephalopat* |
| #16 | brain injury |
| #17 | neuronal injury |
| #18 | neuronal loss |
| #19 | #11 OR #12 OR #13 OR #14 OR #15 OR #16 OR #17 OR #18 |
| #20 | #4 AND #10 |
| #21 | #4 AND #19 |
| #22 | #20 OR #21 |
| Full text search phrase  #22 | (((((((((((((newborn AND (xenon OR argon OR noble gases OR inert gases)) OR (infant AND (xenon OR argon OR noble gases OR inert gases)) OR (pups AND (xenon OR argon OR noble gases OR inert gases)) OR (neonat* AND (xenon OR argon OR noble gases OR inert gases)) OR (perinat* AND (xenon OR argon OR noble gases OR inert gases)) OR (hypoxi* AND (xenon OR argon OR noble gases OR inert gases)) OR (ischemi* AND (xenon OR argon OR noble gases OR inert gases)) OR (ischaemi* AND (xenon OR argon OR noble gases OR inert gases)) OR (asphyxia* AND (xenon OR argon OR noble gases OR inert gases)) OR (encephalopat* AND (xenon OR argon OR noble gases OR inert gases)) OR (brain injury AND (xenon OR argon OR noble gases OR inert gases)) OR (neuronal injury AND (xenon OR argon OR noble gases OR inert gases)) OR (neuronal loss AND (xenon OR argon OR noble gases OR inert gases) |

**Supplementary Table 2. Modified CAMARADES checklist**

| **Study** | **Mitigation of Bias Score** | | | | | | | | | |
| --- | --- | --- | --- | --- | --- | --- | --- | --- | --- | --- |
|  | **Physiol** | **Random** | **Protocol blinding** | **Assessment blinding** | **Power calculation** | **Regulatory compliance** | **Conflict of interests** | **Peer reviewed publication** | **Control of temperature** | **Total score** |
| **Xenon** | **11%** | **72%** | **44%** | **78%** | **5.5%** | **100%** | **83%** | **100%** | **94%** |  |
| Chakkarapani *et al* 2010 | 1 | 1 | 0 | 1 | 0 | 1 | 1 | 1 | 1 | **7** |
| Dingley *et al* 2006 | 0 | 1 | 0 | 1 | 0 | 1 | 0 | 1 | 1 | **5** |
| Faulkner *et al* 2011 | 1 | 1 | 1 | 1 | 1 | 1 | 1 | 1 | 1 | **9** |
| Hobbs *et al* 2008 | 0 | 1 | 1 | 1 | 0 | 1 | 1 | 1 | 1 | **7** |
| Liu *et al* 2015 | 0 | 1 | 1 | 1 | 0 | 1 | 1 | 1 | 1 | **7** |
| Luo *et al* 2008 | 0 | 0 | 0 | 1 | 0 | 1 | 1 | 1 | 1 | **5** |
| Ma *et al* 2006 | 0 | 0 | 0 | 1 | 0 | 1 | 1 | 1 | 1 | **5** |
| Ma *et al* 2005 | 0 | 0 | 0 | 0 | 0 | 1 | 1 | 1 | 1 | **4** |
| Martin *et al* 2007 | 0 | 1 | 1 | 1 | 0 | 1 | 1 | 1 | 1 | **7** |
| Rajakumaraswamy *et al* 2006 | 0 | 0 | 0 | 0 | 0 | 1 | 1 | 1 | 1 | **4** |
| Sabir *et al* 2014 | 0 | 1 | 1 | 1 | 0 | 1 | 1 | 1 | 1 | **7** |
| Sabir *et al* 2016 | 0 | 1 | 1 | 1 | 0 | 1 | 1 | 1 | 1 | **7** |
| Sun *et al* 2023 | 0 | 1 | 0 | 0 | 0 | 1 | 1 | 1 | 1 | **5** |
| Thoresen *et al* 2009 | 0 | 1 | 1 | 1 | 0 | 1 | 1 | 1 | 1 | **7** |
| Yang *et al* 2012 | 0 | 0 | 0 | 1 | 0 | 1 | 1 | 1 | 1 | **5** |
| Zhang *et al* 2020 | 0 | 1 | 0 | 0 | 0 | 1 | 1 | 1 | 1 | **5** |
| Zhang *et al* 2019 | 0 | 1 | 0 | 1 | 0 | 1 | 0 | 1 | 1 | **5** |
| Zhuang *et al* 2012 | 0 | 1 | 1 | 1 | 0 | 1 | 0 | 1 | 0 | **7** |
|  |  |  |  |  |  |  |  |  |  |  |
| **Argon** | **25%** | **100%** | **50%** | **100%** | **25%** | **100%** | **75%** | **100%** | **50%** |  |
| Broad *et al* 2016 | 1 | 1 | 0 | 1 | 1 | 1 | 1 | 1 | 1 | **8** |
| Zhao *et al* 2016^*^ | 0 | 1 | 1 | 1 | 0 | 1 | 1 | 1 | 0 | **6** |
| Zhao *et al* 2016^#.^ | 0 | 1 | 0 | 1 | 0 | 1 | 1 | 1 | 1 | **6** |
| Zhuang *et al* 2012 | 0 | 1 | 1 | 1 | 0 | 1 | 0 | 1 | 0 | **7** |
|  |  |  |  |  |  |  |  |  |  |  |
| **Global** | **13.6%** | **77%** | **45%** | **82%** | **9%** | **100%** | **82%** | **100%** | **86%** |  |

Green = high quality, low risk of bias; Orange = medium quality, medium risk of bias. ^*^Oncotarget v7, p25640-51 ^#^Anesthesiology v125, p180-92

**
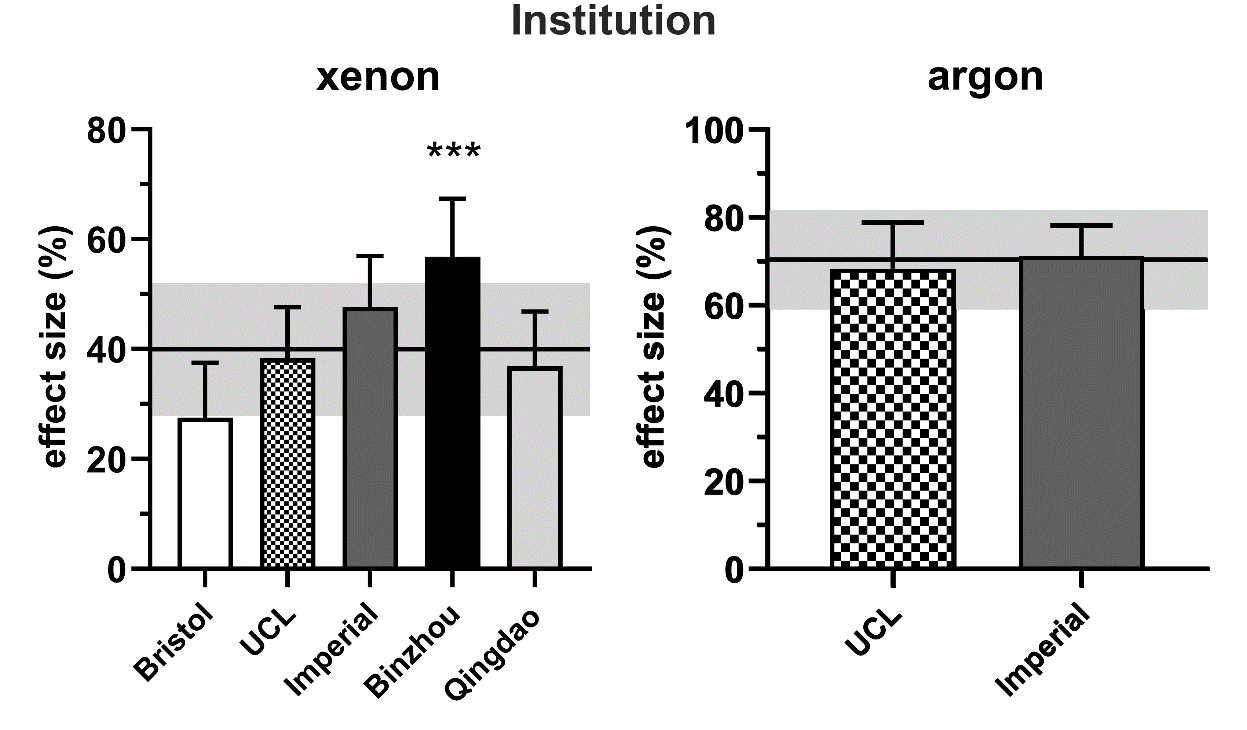
**

**Supplementary Figure 1** Subgroup analysis of neurological outcome effect size comparisons by institution for xenon and argon. Bars are mean values; error bars represent SEM. Differences between subgroups were tested with chi-square test (*** p < 0.001). The overall meta-analysis estimate and 95% CI are indicated by the solid grey line and the light grey shading, respectively.


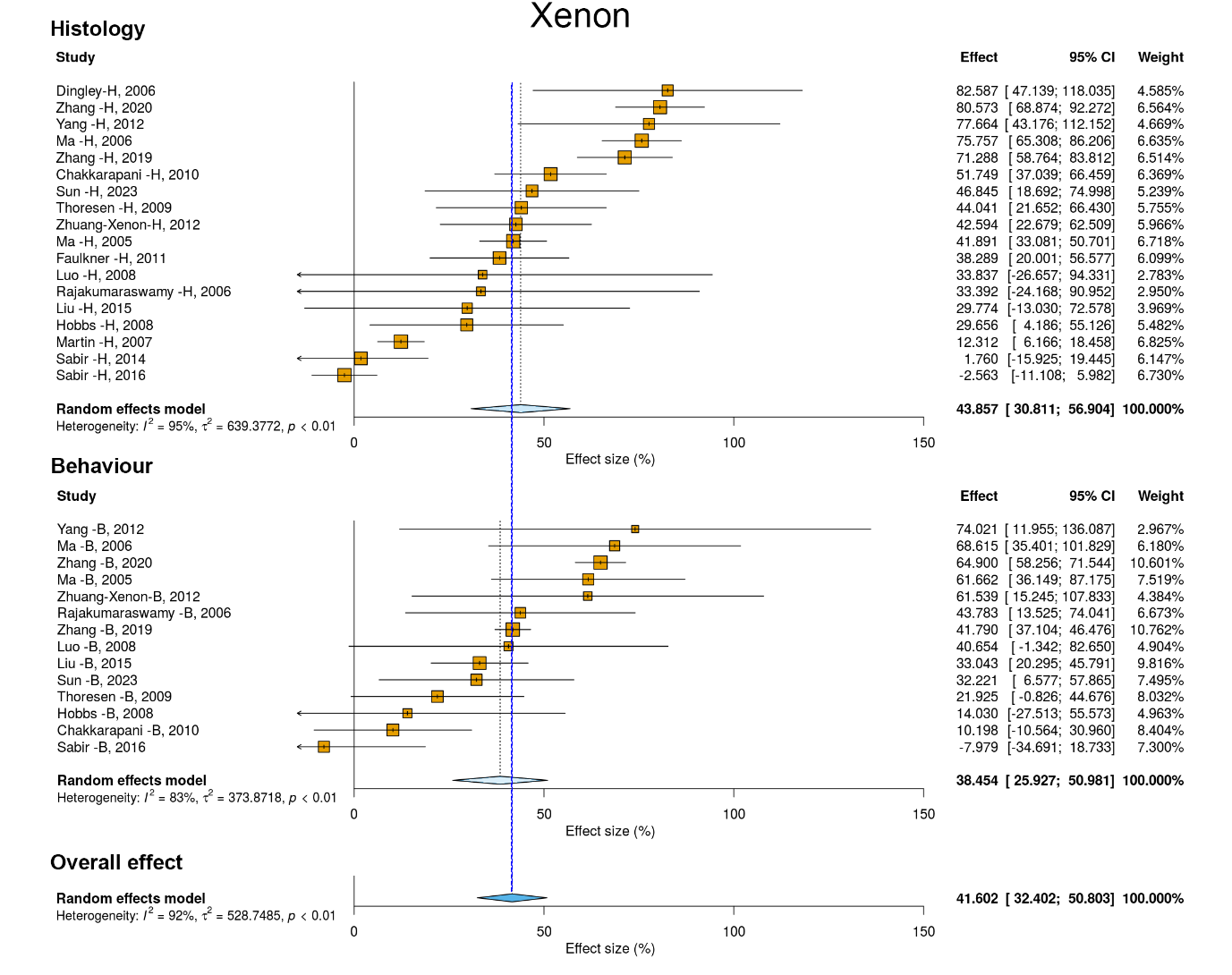


**Supplementary Figure 2.** Stratified meta-analysis of xenon data showing forest plots with estimates of effect sizes for improvement in histological and behavioural outcomes, (Effect size, CI and weight). Positive values represent neuroprotection (improvement). The area of each square is proportional to the study's weight in the meta-analysis. The 95% Cls are shown as horizontal lines. The vertical blue dotted line centered on the dark blue diamond denotes the overall mean effect size. The dotted black lines represent effect sizes for histology, behavour and weight components individually. The 95% CI or overall mean is represented by the width of blue diamond. The first author and date of publication are listed on the left-hand column, while the right-hand column lists the effect size, CI and weighting for each study.

**
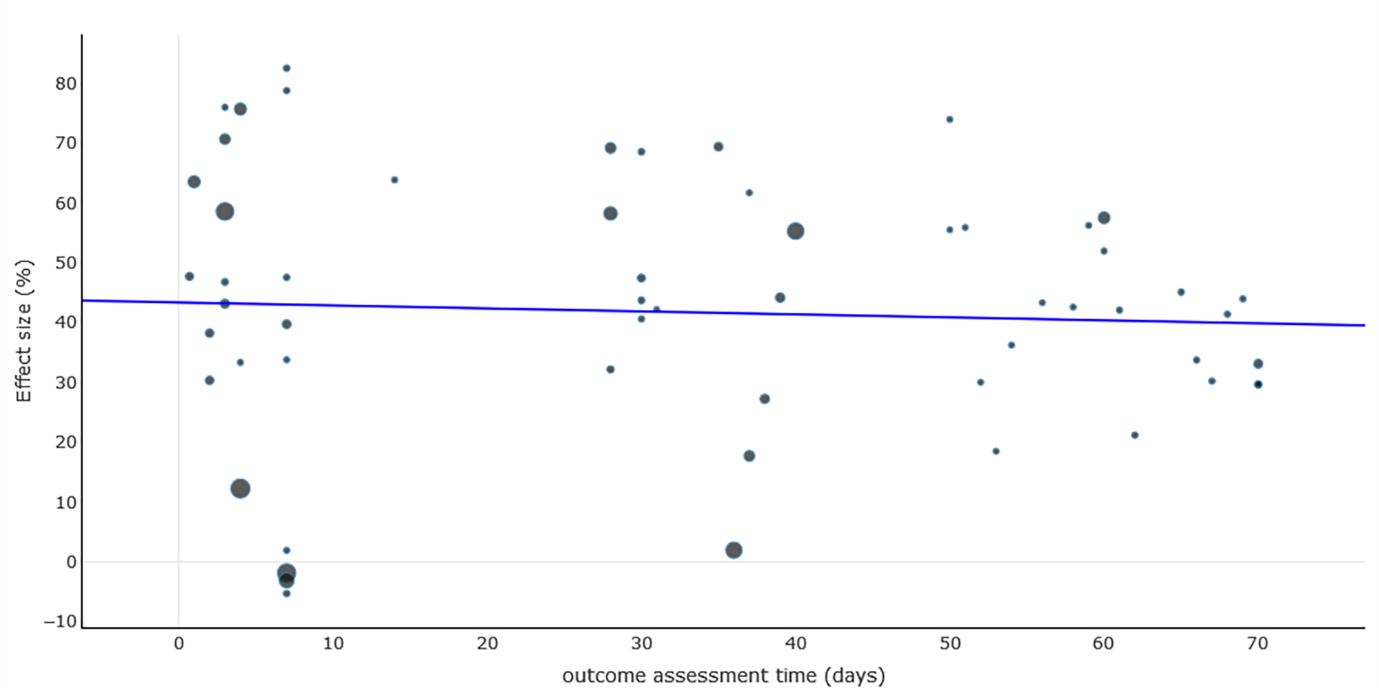
**

**Supplementary Figure 3** Meta-regression of effect sizes for xenon data against time after HI when outcome was assessed. The black circles are the effect sizes for each outcome with the area of the circle proportional to the weighting in the regression (inverse variance). The blue line is the regression line (slope -0.049 ± 0.145 (p=0.73); y-intercept 43.4% ± 5.3% (p<0.001)). The slope of the line was not significantly different to zero.
